# The Testing Effect for Visual Materials Depends on Preexisting Knowledge

**DOI:** 10.1037/xlm0001248

**Published:** 2023-06-08

**Authors:** Catarina S. Ferreira, Maria Wimber

**Affiliations:** 1 School of Psychology and Centre for Human Brain Health (CHBH), University of Birmingham; 2 School of Psychology and Neuroscience and Centre for Cognitive Neuroimaging (CCNi), University of Glasgow

**Keywords:** retrieval-induced enhancement, visual objects, memory consolidation, recollection, episodic memory

## Abstract

Remembering facilitates future remembering. This benefit of practicing by active retrieval, as compared to more passive relearning, is known as the testing effect and is one of the most robust findings in the memory literature. It has typically been assessed using verbal materials such as word pairs, sentences, or educational texts. We here investigate if memory for visual materials equally benefits from retrieval-mediated learning. Based on cognitive and neuroscientific theories, we hypothesize that testing effects will be limited to meaningful visual images that can be related to preexisting knowledge. In a series of four experiments, we systematically varied the type of material (meaningless “squiggle” shapes vs. meaningful object images) and the format of the test used to probe memory (a visually driven alternative forced-choice test vs. a remember/know recognition test). Within each experiment, we assessed the effects of practice type (retrieval or restudy) and the delay of the final test (immediate vs. 1 week) on the resulting practice benefits. Abstract shapes never showed a significant testing benefit, irrespective of test format. Meaningful object images did benefit from testing, particularly at long delays, and with a test format probing the recollective component of recognition memory. Together, our results indicate that retrieval can facilitate the recollection of visual images when they represent meaningful semantic units. This pattern of results is predicted by cognitive and neurobiologically motivated theories proposing that retrieval’s benefits emerge through spreading activation in semantic networks, producing more easily accessible and longer-lasting memory traces.

It is safe to say that most students do not enjoy being tested on course materials. However, research unambiguously shows that testing benefits memory retention. More precisely, actively and repeatedly retrieving newly learned information enhances its long-term retention much more than other types of reexposure, such as rereading or restudying that same information (see Karpicke, 2017; Roediger & Karpicke, 2006a for reviews). This retrieval practice advantage is known as the testing effect. Retrieval benefits are highly robust and most commonly found after a delay between practice and the memory test, while a restudy advantage is sometimes observed when memory is probed shortly after practice (e.g., Roediger & Karpicke, 2006b). Despite being one of the most robust effects in the memory literature, testing effects have largely been demonstrated using verbal materials. We here investigate if and under what conditions this retrieval benefit extends to memory for visual materials.

Being such a well-studied phenomenon, the relative lack of experiments assessing the testing effect using visual materials may be surprising. The vast majority of experiments in the literature used verbal stimuli, such as vocabulary word pairs, sentences, short narratives, or educational texts. A number of studies have attempted to replicate the effect with visuospatial materials. However, with a few exceptions discussed later, they tested participants’ memory for an associated label or name, rather than their memory for the stimuli’s visual features (Carpenter & DeLosh, 2005; Coppens et al., 2011; Jonker et al., 2018; Tse et al., 2010), or they used materials that can easily be verbalized (Carpenter & Kelly, 2012; Carpenter & Pashler, 2007; Guran et al., 2019; Herweg et al., 2018).

Understanding whether the testing effect extends to visual stimuli is a key question to further our understanding of the mechanisms underlying this effect, which is to this day very limited (Karpicke, 2017; Rowland, 2014). Some cognitive theories attribute testing benefits to a deeper, more concept-based processing during retrieval, compared to restudy (Carpenter, 2009, 2011; A. Congleton & Rajaram, 2012; Pyc & Rawson, 2010; Siler & Benjamin, 2020; Verkoeijen et al., 2012). This greater conceptual processing is thought to result from the coactivation of related information during retrieval. Whereas activation during restudy is largely limited to features of the restudied item itself (Carpenter & Yeung, 2017; Sinclair & Barense, 2019), a retrieval cue—due to the imprecise nature of retrieval—is thought to trigger activation that spreads beyond the exact target memory (Antony et al., 2017; Carpenter & Yeung, 2017; Sinclair & Barense, 2019). Coactivated items might then become part of an updated memory, facilitating future access to the target by serving as additional retrieval cues (*elaborative retrieval hypothesis*; Carpenter, 2009) or as a mediating link between cue and target (*mediator effectiveness hypothesis*; Pyc & Rawson, 2010). Congruent with this hypothesis, neuroimaging studies have shown that, compared to restudy, retrieval leads to greater activation of related memories (Ferreira et al., 2019; Jonker et al., 2018). Bringing together cognitive and neurobiologically inspired theories, we recently proposed that retrieval exerts its beneficial effects via a similar mechanism as offline (e.g., sleep-dependent) memory consolidation (Antony et al., 2017). In this framework, retrieval cues trigger the reactivation of a recently learned item in a hippocampal-neocortical network, facilitating the integration of this new item into preexisting neocortical knowledge structures.

The above theories share the assumption that the benefits of retrieval-mediated learning depend on the spread of activation to associated memories. Note, however, that alternative theories, most prominently the episodic context account (Karpicke et al., 2014), can explain many empirical observations by assuming that retrieval reinstates the prior learning context, thereby strengthening links between the retrieved item and its distinct context features, which in turn increases the likelihood of future retrieval (Lehman et al., 2014). We will return to this account in the general discussion. The present research was motivated by explanations of the testing effect based on semantic spreading activation (Antony et al., 2017; Carpenter, 2009; Pyc & Rawson, 2010), and we here asked if preexisting knowledge is a necessary condition for a testing effect to emerge. In other words, can retrieval only enhance meaningful information that can be linked to existing semantics, or can memory for novel, meaningless images also be enhanced via testing?

We conducted four behavioral experiments (Figure 1A) to assess whether memory for visual information can be strengthened via retrieval. In these experiments, participants studied words paired with images, which were either meaningless abstract shapes (*squiggles*; Experiments 1A and 1B) or meaningful objects (e.g., a mug, a key, etc.; Experiments 2A and 2B). Subsequently, half of the participants in each experiment retrieved a subset of the word–image associations, while the other half restudied a subset. Both groups were then tested right after practice (immediate test for half of the items) and a week later (delayed test for the other half). The final test was either a three-alternative forced-choice (3-AFC) memory test (Experiments 1A and 2A) or a remember–know recognition test (Experiments 1B and 2B), both using similar lure images and designed to emphasize recollection of visual image details. Thus, we systematically combined the type of material (meaningful or meaningless) with each final memory test format (3-AFC or remember–know) across the four experiments.[Fig fig1]

Within each experiment, our major dependent variable of interest was the practice benefit, that is, the difference between practiced and nonpracticed items. A testing effect is present if practice benefits are larger in the retrieval compared to the restudy group. We hypothesized that the testing effect would be present in Experiments 2A and 2B that use meaningful object images, but not in Experiments 1A and 1B that use meaningless squiggles. Moreover, we expected the retrieval benefit to be most pronounced in the delayed, compared to the immediate, final memory test, as often found in the testing effect literature (Coppens et al., 2011; Roediger & Karpicke, 2006b; Toppino & Cohen, 2009; van den Broek et al., 2014).

## Experiment 1A

### Method

#### Participants

Forty-eight participants (*M*_age_ = 22.6, *SD* = 3.2, 42 female) took part in this study. Sample size was chosen based on typical samples in previous testing effect studies (Guran et al., 2019; Herweg et al., 2018; Jonker et al., 2018; Sutterer & Awh, 2016; Wing et al., 2013). Participants were randomly assigned to one of two practice conditions, with a total of 24 participants assigned to retrieval and 24 to restudy. The participants were undergraduate or postgraduate students at the University of Birmingham and received either course credits or a monetary reward for their participation. All of them reported normal or corrected-to-normal vision and no history of neurological, psychological, or psychiatric conditions. Before the start of the experiment, participants signed an informed consent form and at the end of the delayed test were debriefed on the aims of the study. The study was approved by the STEM Ethics Committee of the University of Birmingham.

#### Materials and Procedure

**Figure 1 fig1:**
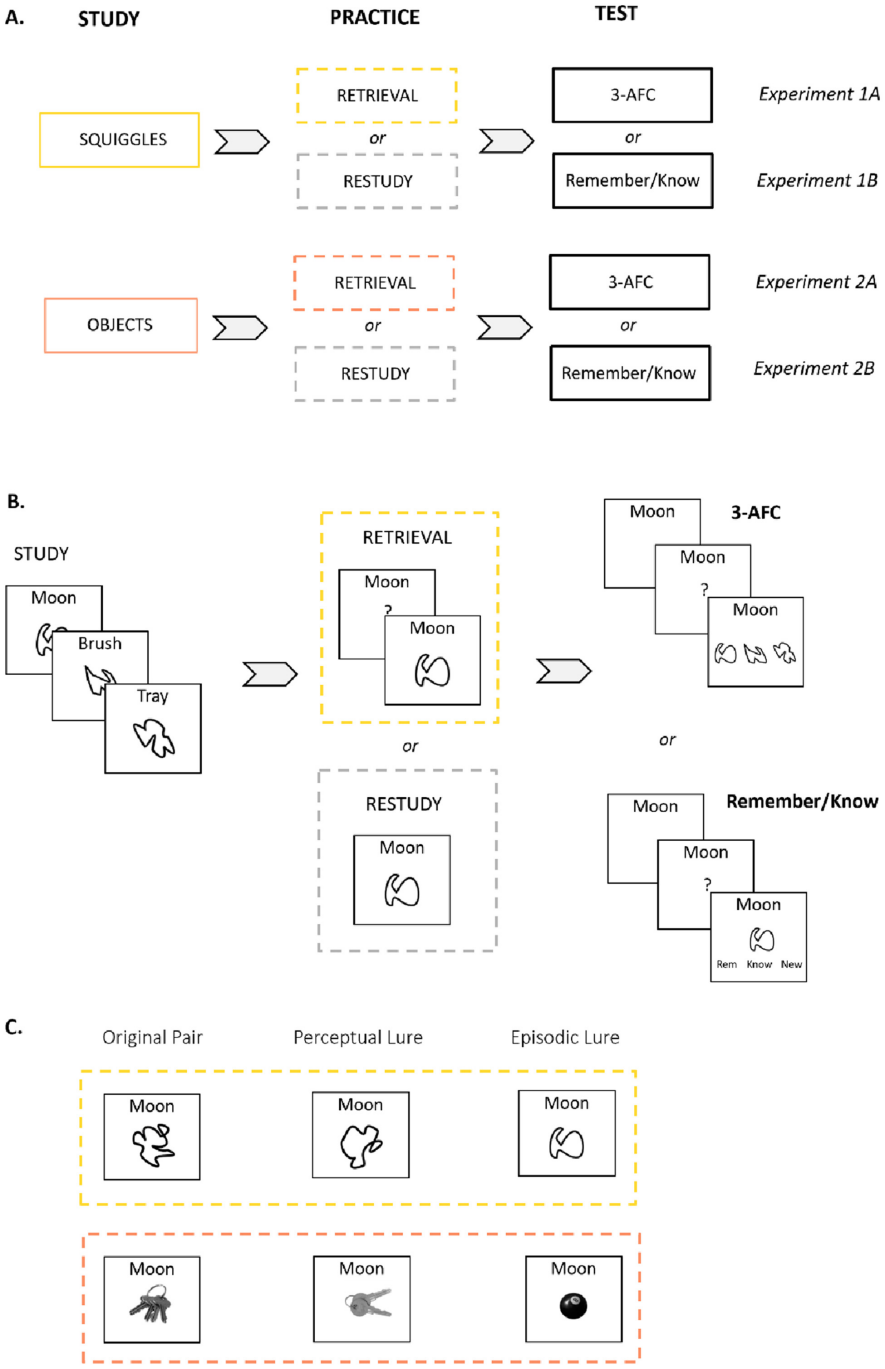
Overview and General Procedure of the Four Experiments and Examples of the Perceptual and Episodic Lures Used in Experiments 1B and 2B *Note*. (A) Overview of the four experiments and how they vary across subjects. (B) General procedure across the four experiments. participants studied word-image pairs that were then either retrieved or restudied. The final test in Experiments 1A and 2A was a three-alternative forced-choice test, whereas Experiments 1B and 2B used an associative recognition test including remember/know judgments. (C) Examples of the perceptual and episodic lures used in Experiments 1B (yellow rectangle) and 2B (orange rectangle). 3-AFC = three-alternative forced-choice. See the online article for the color version of this figure.

In this experiment, we used 40 (36 critical, four used for a familiarization task) black abstract shapes (*squiggles*; Figure 1C, upper yellow box), presented on white backgrounds. These stimuli were kindly shared by Groh-Bordin et al. (2006) and can be found at https://osf.io/6zf3t/ (Ferreira & Wimber, 2021). Each *squiggle* was randomly paired with a word. Words were drawn from the MRC psycholinguistic database (https://websites.psychology.uwa.edu.au/school/MRCDatabase/uwa_mrc.htm) and had similar values of imageability (*M* = 567.9, *SD* = 10.9), concreteness (*M* = 583.7, *SD* = 30.7), and meaningfulness (*M* = 447.4, *SD* = 31.1; all three scales measured in a range of 100–700).

The experiment consisted of four main stages: study, practice (retrieval or restudy, manipulated between subjects), immediate memory test, and delayed memory test (Figure 1A and B). The immediate test took place immediately after practice, whereas the delayed test took place after 7 days.

During study, participants saw a word in black font at the top of the screen, with a *squiggle* (4.8 × 6.4 cm) below for 7 s. Participants were instructed to link the word and the squiggle as well as they could, as they would be tested on the pairs later. In addition to memorizing the pairs, participants were asked to press a key to indicate whether they found it easy or hard to link the word and the *squiggle* together (left for easy, right for hard). Since these meaningless shapes are difficult to memorize (as revealed in a pilot study), each pair was repeated twice during the study phase. The order of stimulus presentation was randomized, but all the stimuli were presented once before repeating again in a new random order.

After study, a quick familiarization phase took place, to assess whether participants were paying attention to the pairs and to prepare them for what would be the format of the final test. This quick test also provided a break between study and practice, the longest phases of the experiment. During this familiarization task, participants saw four of the previously studied pairs. They were first presented with a word at the top of the screen and asked to think back to the *squiggle* associated with this word. After 4 s, a question mark appeared below the word, and participants were asked to indicate whether they thought they remembered the correct item (left arrow key) or not (right arrow key). Upon response, three different *squiggles* (all previously studied) appeared below the word, and participants were asked to pick the one that had been paired with that particular word by pressing one of the arrow keys on the keyboard (left for the leftmost stimulus on the screen, down for the middle stimulus, and right for the rightmost one). This 3-AFC screen disappeared upon participants’ response or after 4 s. The stimuli presented in the familiarization task were not shown again in the remaining parts of the experiment.

After familiarization, participants were informed that they would now have an opportunity to practice some of the pairs. Participants in the restudy condition (24/48) were told they would see some of the previously studied pairs and should use this reexposure as a chance to encode them again. The pairs were shown in the same way they had during study for 7.5 s, and participants had to indicate if they still found it easy or hard to link the pair, using the same response keys as in the study phase. Participants in the retrieval condition (24/48) were asked to actively bring the *squiggles* back to mind, upon being prompted with the word as a retrieval cue. The word appeared at the top of the screen with a question mark below for 5 s, during which participants were instructed to vividly bring the *squiggle* back to mind. The corresponding *squiggle* was then presented for 2.5 s to provide feedback. In both conditions, 24 of the 36 critical pairs were presented twice for practice. Stimuli were presented in random order, but all 24 items were presented once before repeating again in a new random order.

The remaining 12 pairs were not practiced and were used as baseline items to assess memory performance without further practice. This baseline measure was included to account for random variability in memory performance between participants and between the restudy and retrieval groups. Reducing such random noise is particularly relevant for the delayed test in our experiment, where differential forgetting rates are likely to increase variance in performance. Memory accuracy for nonpracticed baseline items was subtracted from accuracy for practiced items (see Statistical Analyses section), yielding a practice benefit for each participant that could then be compared between the two groups and between the immediate and delayed test.

The assignment of each word–image pair to practice or baseline was counterbalanced across participants. Note that both conditions (retrieval and restudy) were equated for overall practice time and were very similar, with the key difference that participants in the retrieval condition had to consciously bring the correct *squiggle* back to mind whereas participants in the restudy condition were presented with the complete pair.

Pairs were pseudorandomly allocated to the immediate or delayed test, so that half of all the pairs (12 practiced and six baseline) were tested immediately after practice, whereas the other half were tested 7 days later, all in random order. Other than that, the two tests were identical and followed the exact same procedure as the familiarization phase.

### Statistical Analyses

The raw data, as well as the averaged data used in all the analyses throughout the manuscript, are available at https://osf.io/6zf3t/. All statistical analyses used practice benefits (accuracy for practiced minus nonpracticed baseline items) as the dependent variable. Since testing effects are typically found after extended delays between practice and final test, we were particularly interested in retrieval benefits at a long delay. Accordingly, in all four experiments of this study, including the present one, we initially conducted one planned comparison, which was a one-tailed independent *t* test comparing the practice benefits between the retrieval and restudy group on the delayed test.

We were additionally interested in whether there was a shift from a restudy benefit in the immediate test to a retrieval benefit in the delayed test, as previously reported in the testing effect literature (e.g., Roediger & Karpicke, 2006b). To assess this Practice Type × Delay interaction, we ran a 2 × 2 mixed analysis of variance (ANOVA) with practice benefit as the dependent variable, and factors practice type (retrieval vs. restudy; manipulated between subjects) and delay (immediate vs. delayed test; manipulated within subjects). Significant effects in this ANOVA were then further assessed in two-tailed post hoc *t* tests.

### Results

**Figure 2 fig2:**
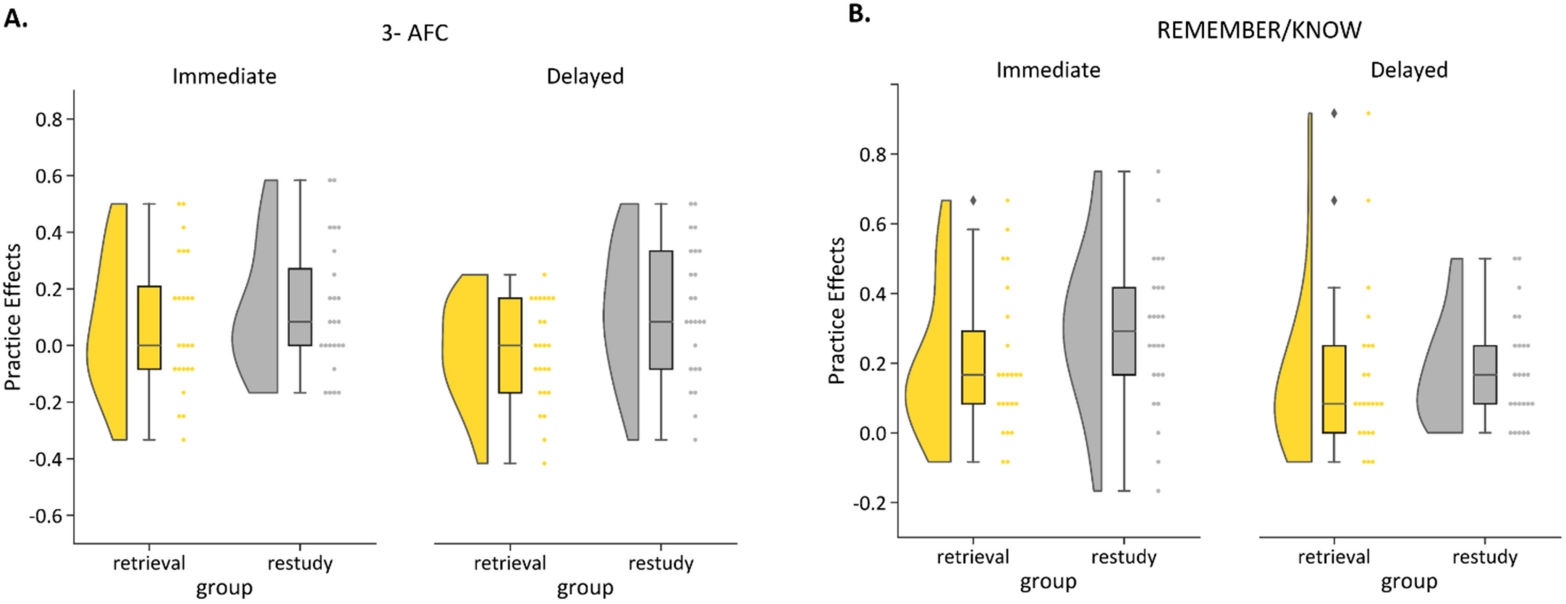
Practice Effects (Accuracy for Practiced Minus Nonpracticed Baseline Pairs) in Experiments 1A (Panel A) and 1B (Panel B), Both Using Nonmeaningful Squiggle Images *Note*. Colored rainclouds represent practice benefits for retrieved items, and gray rainclouds represent practice benefits for restudied items, in an immediate (left of the graphs) and delayed (right of the graphs) memory test. 3-AFC = three-alternative forced-choice. See the online article for the color version of this figure.

The results of Experiment 1A are depicted in Figure 2A, showing practice effects for the squiggle images depending on the type of practice and delay (see Table 1 for results breaking down performance for practiced and baseline items). The one planned comparison of interest revealed no significant benefit of retrieval over restudy on the delayed memory test, *t*(46) = −2.44, *p* = .99. In fact, a significant effect in the opposite direction was found (see post hoc tests below).[Fig fig2][Table tbl1]

Results from the mixed ANOVA revealed no significant Practice Type × Delay interaction, *F*(1, 46) = 1.42, *p* = .239. There was no main effect of delay, *F*(1, 46) = 1.62, *p* = .210, but we did find a significant main effect of practice type, *F*(1, 46) = 4.08, *p* = .049, η_*p*_^2^ = .08. Post hoc comparisons revealed that participants in the restudy group showed significantly larger practice benefits, across immediate and delayed test (*M* = 0.12, *SD* = 0.23), than those in the retrieval group, *M* = 0.03, *SD* = 0.22; *t*(94) = −2.14, *p* = .035. This restudy advantage was statistically significant only on the delayed test, immediate test: *t*(46) = −.696, *p* = .490; delayed test: *t*(46) = −2.44, *p* = .019.

### Discussion

In Experiment 1A, our main comparison of interest revealed no retrieval benefit for novel, meaningless shapes. In contrast, we found a reversal of the testing effect, with restudied items benefiting significantly more from practice than retrieved items at a longer delay.

There are several possibilities as to why a testing effect was not found here. One is that, as hypothesized, retrieval does not enhance memory for novel visual stimuli that have no preexisting representation in semantic memory. In fact, not only was no testing effect found for these meaningless squiggle images, but restudy seemed to benefit their long-term retention to a greater extent. This tendency for a restudy advantage was present at both delays, though only significant at the 1-week delay, with no interaction between practice type and delay. These findings are consistent with theories ascribing testing effects to the coactivation of semantically related information during retrieval (Antony et al., 2017; Carpenter, 2009, 2011; Pyc & Rawson, 2010; Sinclair & Barense, 2019). If the to-be-retrieved material has no existing semantic representation, spreading activation to similar information is not possible.

An alternative explanation is that retrieval practice for these meaningless shapes was simply too difficult. If participants in the retrieval group were largely unsuccessful at bringing back to mind and visualizing the correct items, this could potentially eliminate any practice benefits, and even make restudy the more advantageous rehearsal strategy. For example, previous work suggests that retrieval practice leads to substantial strengthening of only those items that are successfully recalled during practice. Restudy, by contrast, moderately strengthens all items uniformly (Kornell et al., 2011; van den Broek et al., 2014). The bifurcation of the item strength distribution caused by retrieval practice can explain why restudy sometimes outperforms retrieval on immediate tests: the moderate strength of restudy items is sufficient to support these items’ recall when little forgetting has happened. However, retrieval will outperform restudy on delayed tests, where forgetting has pushed most restudy items below the accessibility threshold, while the subset of items that were successfully retrieval practiced will remain accessible (Rowland & DeLosh, 2015). Note that this account does not provide a mechanistic explanation for the different processes underling restudy and retrieval practice. It does, however, predict that retrieval benefits are limited to items successfully retrieved, or corrected by feedback (Rowland & DeLosh, 2015). Such failure to retrieve, however, is unlikely to have caused our pattern of results. First, feedback was provided for 2.5 s on each retrieval trial, exposing participants to the correct item even if they had not been able to retrieve it. Secondly, baseline performance levels were comparable between the retrieval and restudy group on the immediate final test, and even numerically higher in the retrieval group on the delayed test (see Table 1), speaking against an effect of low retrieval practice success.

Finally, a third possibility that could account for our results is that the final memory test used in this experiment was not sensitive to retrieval benefits. As Chan and McDermott (2007) have pointed out, testing effects are evident only when the final test specifically encourages controlled retrieval of the studied items. Across four experiments, these authors and others (Pu & Tse, 2014; Verkoeijen et al., 2011) demonstrated that recognition tests that rely heavily on familiarity often fail to detect any differences between retrieval and restudy conditions, with differences becoming evident, however, when final memory tests specifically probe recollection.

While previous research suggests that AFC tests depend on recollection (e.g., Cook et al., 2005; Kroll et al., 2002; Khoe et al., 2000), especially when using familiar lures as in the present design (Mayes et al., 2002; Migo et al., 2009), others have argued that discrimination in these tests can be achieved on the basis of familiarity (e.g., Bastin & Van der Linden, 2003).

To minimize the contribution of familiarity and isolate the recollective component of memory retention on the final test, we conducted the same experiment again, now using a remember/know associative recognition procedure as the final memory test instead of the 3-AFC. Associative recognition tests are thought to depend strongly on recollection (Hockley & Consoli, 1999), especially when participants are asked to judge the oldness of stimuli that are all familiar but presented in a rearranged fashion (e.g., reshuffled study pairs). In this case, familiarity is less useful in supporting recognition (since all of the items are equally familiar; Yonelinas et al., 2010), and the rejection of rearranged pairs requires recollection (Castel & Craik, 2003). However, associative recognition has also been shown to be subject to low-level perceptual influences (Goshen-Gottstein & Moscovitch, 1995). To account for this, we added remember/know judgments to our final test to specifically isolate the recollection component of the recognition process. In this procedure, a “remember” response is thought to reflect recollection processes, whereas “know” responses should be based on familiarity (Gardiner, 1988; Migo et al., 2012; Tulving, 1985). In Experiment 1B (and also Experiment 2B), we thus counted only correct “remember” responses as successfully retrieved, allowing us to isolate the benefits of retrieval and restudy practice specifically on recollection-based memory (for analyses including “know” responses, see pages 1 and 2 in the online supplemental materials).

## Experiment 1B

### Method

#### Participants

A novel sample of 48 participants took part in this study. Participants were randomly assigned to the two experimental conditions. One of the participants failed to show up for session 2, and their data were thus excluded from any further analyses. Of the remaining 47 participants (*M*_age_ = 19.1, *SD* = 0.8, 41 female), 23 performed retrieval practice and 24 performed restudy practice. Participants were undergraduate or postgraduate students at the University of Birmingham and received either course credits or a monetary reward for their participation. All of them reported normal or corrected-to-normal vision and no history of neurological, psychological, or psychiatric conditions. Before the start of the experiment, participants signed an informed consent form and at the end of the delayed test were debriefed on the aims of the study. The study was approved by the STEM Ethics Committee of the University of Birmingham.

#### Materials and Procedure

The main difference between Experiments 1A and 1B was the final test, where an associative recognition test including remember/know judgments was used instead of the 3-AFC test. For the associative recognition test, additional *squiggle* images were selected as lure images to be shown on repaired trials. Of the 40 *squiggles* shown at study, 20 were knotted (their lines crossed at one point of the drawing) whereas the other 20 were simple *squiggles* (i.e., not knotted—no lines crossed; see upper yellow box in Figure 1C). This feature was used to select perceptually similar lures for the final test (see below). Other than that, the study phase was the same as in Experiment 1A.

The familiarization phase served again as a preparation for the final tests. As before, participants saw the cue word for 4 s and were asked to think back to the associated *squiggle*. Then, a question mark appeared below, and participants were asked to report by button press whether they remembered the correct item or not. They were then presented with an item below the word. Participants had to indicate if the item had originally been presented with that same word or not (see below). The item was on screen until response or up to a maximum of 4 s.

The *squiggle* shown, together with a word, in the familiarization task and the final tests could be (a) *exactly* the same that had been studied in the first phase of the experiment (original pairs), (b) a *squiggle* that had never been seen before, but was perceptually similar to the studied one (perceptual lures) or (c) a *squiggle* and a word that had both been previously seen in the experiment, but had not been paired together (episodic lures; see Figure 1C). Knotted squiggles served as perceptual lures for other knotted squiggles as did simple for simple. The participants’ task was to decide if a given pairing was old (intact) or new (repaired). They were made aware of the different types of pairs and were instructed to respond with “old” *only* to the original pairs and “new” to the two types of repaired probes (i.e., perceptual and episodic lures). Moreover, if the item was old, they were asked to indicate whether they *remembered* (that is, they distinctively remembered seeing the item and the word paired together during the study phase of the experiment) or *knew* it (had the feeling they had seen the pair before, without precise recollection). Participants pressed the left arrow on the keyboard for “old-remember,” the down arrow for “old-know” and the right arrow for “new.” These prompts were shown at the bottom of the screen, below the *squiggle*, in the left, middle, and right positions, respectively (Figure 1B). In the familiarization phase, four original pairs, one episodic lure, and one perceptual lure were shown in random order.

After the familiarization phase, participants performed the practice phase twice. The retrieval and restudy conditions were identical to Experiment 1A. The only difference was that we asked participants in the retrieval condition for a subjective memory response before the probe *squiggle* appeared on the screen by pressing the left button if they thought they remembered the correct item and pressing the right button if they did not remember the item. This button press was included to provide us with an indication of memory success, even though subjective, which was not available for Experiment 1A. In pages 3–5, the online supplemental materials report these subjective judgments. As in the previous experiment, all items were presented once in random order before repeating again, in a new random order. Assignment of each pair to practice or baseline, and of each *squiggle* to target or lure, was counterbalanced across participants.

The final tests (immediate and delayed) followed the same procedure as the familiarization phase. Pairs were pseudorandomly chosen within each participant’s learning set to be tested immediately or after a week, so that at each test stage, 18 original pairs, 18 episodic lures, and 18 perceptual lures were tested. Of these, 12 were previously practiced items (or lures of practiced items) and six were baseline items (or lures of baseline items). Half were knotted and half were simple *squiggles*.

In this experiment, we used two types of lure items: perceptual and episodic. If retrieval strengthens the meaningful aspects of a memory (Ferreira et al., 2019; Lifanov et al., 2021), this might come at the cost of perceptual detail (Lifanov et al., 2021) and increase false alarms to perceptually similar lures (Lee et al., 2017). For instance, repeatedly retrieving the image of a set of keys (see example in Figure 1) will presumably activate and strengthen the existing concept “key” (Antony et al., 2017) at the expense of finer perceptual details of the specific set of keys that had been studied (see Lee et al., 2017). Accordingly, we additionally hypothesized that in our experiments using the associative recognition final memory test (Experiments 1B and 2B), retrieval (compared to restudy) would specifically increase perceptual, but not episodic, false alarms.

Finding perceptual lures in Experiment 2B, where concrete objects were presented as stimuli (see below), was relatively straightforward. For example, we selected a set of keys that is perceptually similar to the target set of keys, but not the same (see Figure 1C). For the present experiment using abstract *squiggle* stimuli, the selection of lure images is more difficult. To keep Experiments 1B and 2B as coherent as possible, we still aimed to approximate the perceptual lure manipulation here. Stimuli in the present experiment included knotted and simple *squiggles* (see Figure 1C for examples), and we used these two categories to draw perceptual lures from the same category as the target *squiggle*; that is, if the target was a knotted *squiggle*, so was the perceptual lure, while simple *squiggles* were used as lures for simple target *squiggles*.

### Statistical Analyses

Like in Experiment 1A, our dependent variable was practice effects, calculated here as the proportion of original pairs that participants correctly recollected (old-remember responses) after practice compared to no practice. As mentioned earlier, our aim was to isolate the effects of testing on recollection, and we therefore only counted old-remember responses as correctly retrieved to obtain a maximally pure measure of recollection (Gardiner, 1988; Tulving, 1985). Results using old-know and all old responses collapsed are reported in pages 1 and 2 of the online supplemental materials. Briefly, these analyses showed a significant effect of delay (where practice benefits were more pronounced in the delayed test) but no other significant effects. These results should be interpreted with caution, given the low number of “know” responses.

Consistent with the previous experiment, we first conducted a targeted one-tailed *t* test comparing the practice benefits (old-remember responses to practiced—baseline items) in the retrieval and restudy groups on the final delayed test. We then ran a 2 × 2 mixed ANOVA with factors practice type (retrieval vs. restudy; manipulated between subjects) and delay (immediate vs. delayed test; manipulated within subjects). Significant results from the ANOVA were further assessed in two-tailed post hoc comparisons.

Finally, we analyzed the proportion of false alarms to perceptual and episodic lures. Because participants rarely gave a “remember” response to new pairings, “remember” and “know” false alarms were collapsed for these analyses. We analyzed old responses to lures of practiced pairs *minus* old responses to lures of baseline items to parallel all other analyses. We were particularly interested if retrieval, compared to restudy, would increase the proportion of perceptual false alarms (Lee et al., 2017).

### Results

Practice effects in Experiment 1B are depicted in Figure 2B, and Table 1 shows mean accuracies separately for practiced and baseline items. The main planned comparison showed no significant retrieval advantage for *squiggles* in the delayed test, *t*(45) = −.232, *p* = .59. Moreover, there was no significant Practice × Delay interaction, *F*(1, 45) = .913, *p* = .344, nor a main effect of practice, *F*(1, 45) = 1.40, *p* = .243, or delay, *F*(1, 45) = 3.09, *p* = .085. No further post hoc tests were thus conducted on the practice benefits.

Analyzing false alarm rates, we found no differences in perceptual false alarms (old responses to perceptual lures of practiced minus of baseline items) between the retrieval and restudy groups on the delayed test, *t*(45) = .926, *p* = .82. Additionally, the 2 × 2 ANOVA indicated no significant interaction between practice type and delay, *F*(1, 45) = .008, *p* = .93, nor a main effect of practice, *F*(1, 45) = 2.34, *p* = .133. There was, however a main effect of delay, *F*(1, 45) = 10.67, *p* = .002, with participants showing a larger practice-related increase in perceptual false alarms on the delayed test compared to the immediate test when collapsing across both groups, *M*_imm_ = −.097, *SD*_imm_ = 0.18; *M*_del_ = .034, *SD*_del_ = 0.18; *t*(46) = −3.304, *p* = .002. No significant effects were found when using false alarms to episodic lures (old responses to episodic lures of practiced minus baseline items) as the dependent variable for any of the planned analyses, *t* test on delayed test: *t*(45) = 1.17, *p* = .88; Delay × Practice interaction: *F*(1, 45) = 1.97, *p* = .17; main effect of practice: *F*(1, 45) = .083, *p* = .74; main effect of delay: *F*(1, 45) = .017, *p* = .90.

### Discussion

Experiment 1B again provided no evidence for a testing effect when using meaningless squiggle images. In contrast with Experiment 1A, we did not find a significant reversal of the testing effect in this study either, although numerically, practice benefits where still higher for the restudy condition (see Table 1). Together with Experiment 1A, this pattern of results suggests that retrieval does not enhance memory for images that have no preexisting semantic representation, irrespective of the final test format. The finding is congruent with predominant theories of the testing effect suggesting that spreading activation in semantic networks plays a central role in producing retrieval’s benefits on long-term retention (Antony et al., 2017; Carpenter, 2011; Ritvo et al., 2019; Sinclair & Barense, 2019): novel, meaningless materials can be assumed to preclude such spread of activation due to a lack of a preexisting associative network, resulting in no testing effect. Instead, restudy can be beneficial in such situations, allowing for additional exposure to the novel materials.

It should be noted, however, that the null findings from Experiment 1B in themselves do not provide strong evidence for or against any theory. If the lack of preexisting knowledge explains the absence of a testing effect in our first two experiments, we should expect a change in direction of the practice benefits when the target images depict meaningful objects, with a clear testing effect emerging on the delayed test.

We thus conducted two further experiments, replacing the abstract *squiggles* with concrete nameable objects. In Experiment 2A, we used a 3-AFC final memory test, whereas in Experiment 2B, participants’ memory was probed with an associative recognition test including remember/know judgments, mirroring Experiments 1A and 1B, respectively. We hypothesized that a retrieval-induced enhancement should be evident particularly in Experiment 2B, where the memory test specifically probes recollection, replicating previous work (Chan & McDermott, 2007; Pu & Tse, 2014; Verkoeijen et al., 2011).

## Experiment 2A

### Method

#### Participants

A new sample of 48 participants (*M*_age_ = 19.2, *SD* = 1.13, 46 female) took part in this study. As in the previous experiments, each participant was randomly assigned to one of the two practice conditions (24 assigned to retrieval and 24 to restudy). Participants were undergraduate or postgraduate students at the University of Birmingham and received either course credits or a monetary reward for their participation. All of them reported normal or corrected-to-normal vision and no history of neurological, psychological, or psychiatric conditions. Before starting the experiment, participants signed an informed consent form and at the end of the experiment were debriefed on the aims of the study. The study was approved by the STEM Ethics Committee of the University of Birmingham.

#### Materials and Procedure

For this experiment, unique nameable objects were chosen as stimuli to be paired with words. The objects were chosen from the Bank of Standardized Stimuli (BOSS; Brodeur et al., 2014; https://sites.google.com/site/bosstimuli/) and modified to grayscale (Figure 1C, orange square).

This experiment followed the same procedure as Experiment 1A. However, to adjust the level of difficulty of the task (nameable objects are easier to remember than abstract shapes, as revealed in a pilot study), in this experiment, there was only one study cycle instead of two, and the total number of stimuli was increased. Participants studied a total of 76 word–object pairs. The cue words had similar levels of imageability (*M* = 564, *SD* = 8.7), concreteness (*M* = 582.8, *SD* = 28.2), and meaningfulness (*M* = 443.4, *SD* = 31.51), as in the previous experiments. Four of the 76 words were used in the familiarization phase and not seen again throughout the experiment. From the remaining 72, 48 were used as part of the retrieval/restudy pairs, and 24 in baseline pairs. As in the previous experiments, the assignment of each pair to item type (practiced or baseline) was counterbalanced across participants. Pairs were pseudorandomly assigned to the immediate or delayed test, so that each final test, immediate and delayed, assessed memory for 24 of the practiced items and for 12 of the baseline ones.

### Statistical Analyses

Statistical analyses were conducted in the same fashion as Experiment 1A: we first conducted a one-tailed independent *t* test, comparing practice benefits (practiced minus baseline) for retrieval versus restudy on the delayed test, where testing effects are typically observed. We then computed a 2 × 2 mixed ANOVA on practice benefits, with factors practice type (retrieval vs. restudy; manipulated between subjects) and delay (immediate vs. delayed test; manipulated within subjects). Significant results from the ANOVA were further assessed in two-tailed post hoc *t* tests.

### Results

**Figure 3 fig3:**
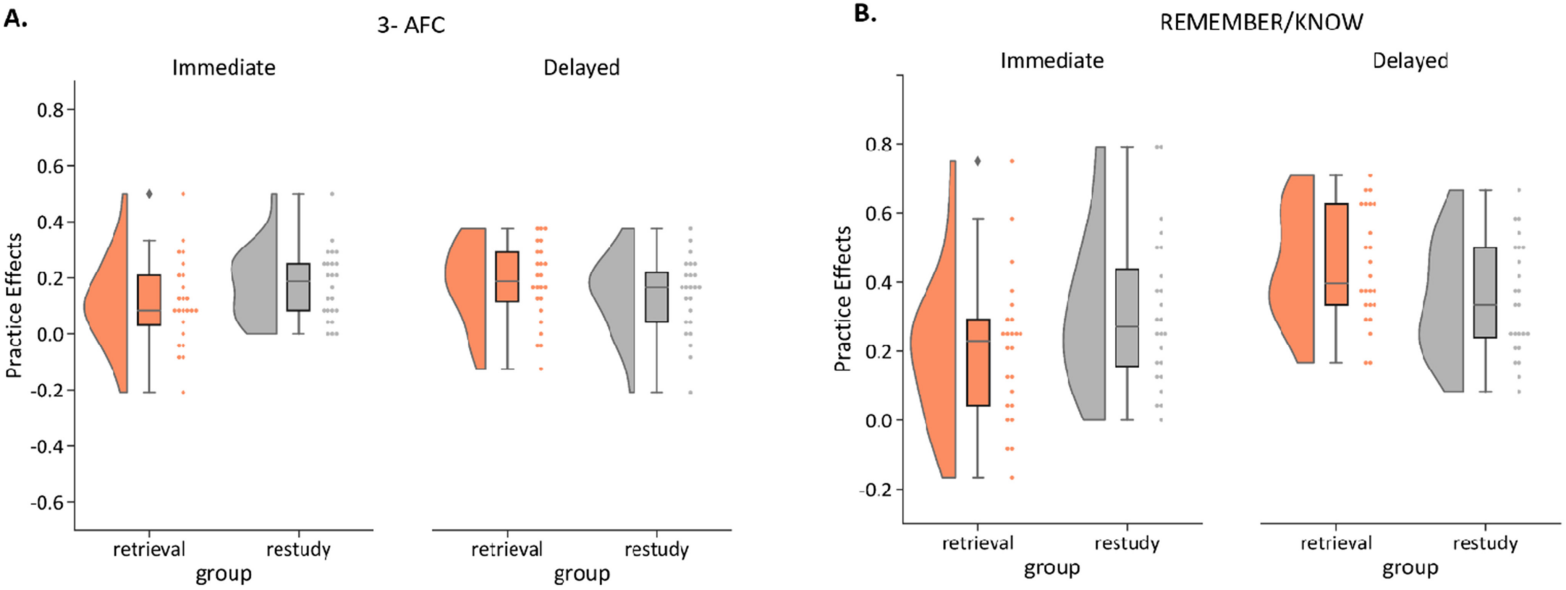
Practice Effects (Accuracy for Practiced Minus Baseline Pairs) in Experiments 2A (Panel A) and 2B (Panel B) *Note*. Colored rainclouds represent practice benefits for retrieved items, and gray rainclouds represent practice benefits for restudied items, in an immediate (left of the graph) and delayed (right of the graph) memory test. 3-AFC = three-alternative forced-choice. See the online article for the color version of this figure.

The results of Experiment 2A (practice effects) are depicted in Figure 3A (see Table 2 for accuracies separately for practiced and baseline items). The planned comparison of practice benefits on the delayed test showed no significant retrieval advantage, *t*(46) = 1.11, *p* = .136.[Fig fig3][Table tbl2]

We found, however, a significant Practice × Delay interaction, *F*(1, 46) = 4.71, *p* = .035, η_*p*_^2^ = .09. In the immediate test, participants in the restudy condition showed larger practice effects (*M* = 0.17, *SD* = 0.13) than those in the retrieval condition (*M* = 0.11, *SD* = 0.16), and this pattern was numerically reversed on the delayed test (*M*_restudy_ = 0.14, *SD*_restudy_ = 0.14; *M*_retrieval_ = 0.18, *SD*_retrieval_ = 0.14). Post hoc comparisons between groups did, however, not reach statistical significance; immediate test: *t*(46) = −1.48, *p* = .15; see planned comparison above for delayed test. Additionally, there was no significant main effect of practice, *F*(1, 46) = .056, *p* = .814, nor a significant main effect of delay, *F*(1, 46) = .531, *p* = .470.

### Discussion

In this experiment we found a pattern of results that qualitatively matches the one commonly reported in the testing effect literature (Roediger & Karpicke, 2006b): while restudy led to numerically larger benefits than retrieval in the immediate test, this pattern was reversed in the delayed test, as indicated by a significant Group × Delay interaction. Although these results are in line with a testing effect for nameable objects, they should be interpreted with caution, since when comparing the two conditions directly in post hoc tests, neither the restudy advantage on the immediate test, nor the retrieval advantage on the delayed test, were statistically robust.

It is possible that retrieval simply does not enhance memory for visual stimuli, even when they are meaningful and nameable. This seems unlikely, however, given that a number of previous studies have reported a testing effect for meaningful visual materials (Carpenter & Kelly, 2012; Carpenter & Pashler, 2007; Guran et al., 2019; Herweg et al., 2018). Another possibility is that the 3-AFC test format used here introduces noise by inflating guessing levels. To equate familiarity between the three alternative choices, we used lure images that were targets on other trials, and participants may have been able to reject them on that basis. We, therefore, added a forth experiment, again using meaningful object images, but probing memory with an associative recognition test with a remember/know procedure, which we used to isolate the recollective component that presumably benefits most strongly from retrieval practice according to previous work (Chan & McDermott, 2007; Pu & Tse, 2014; Verkoeijen et al., 2011).

## Experiment 2B

### Method

#### Participants

Another 48 participants took part in this study. Due to data loss during digitization, demographic data are only available for half of the sample. For the participants whose demographic data are available, mean age was 21.6 years old (*SD* = 3.6; 22 out of 24 female). As in the previous experiments, participants were randomly assigned to a retrieval or a restudy condition (24 participants per condition). All participants were undergraduate or postgraduate students at the University of Birmingham and received either course credits or a monetary reward for their participation. All of them reported normal or corrected-to-normal vision and no history of neurological or psychiatric conditions. Prior to the start of the experiment, participants signed an informed consent form and at the end of the delayed test were debriefed on the aims of the study. The study was approved by the STEM Ethics Committee of the University of Birmingham.

#### Materials and Procedure

The same materials from Experiment 2A were used in Experiment 2B. This experiment followed a similar procedure to that of Experiment 1B, the only differences being the number of study cycles (only one instead of two) and the number of stimuli. The same number of stimuli as in Experiment 2A was used (72 critical objects), with additional lures added to this image pool. Episodic lures were images that had been learned and practiced throughout the experiment but were paired with a different word during the final test (i.e., recombined pairs). Perceptual lures were new, unstudied images that resembled the target item for a given trial (see Figure 1C). Many of these images were chosen through Google image searches, as not every stimulus used in Experiment 2A had a perceptually similar image in the BOSS database. Pair assignment to item type (practice or baseline) was counterbalanced across participants, as was the assignment of each image to target or lure. Pairs were pseudorandomly assigned to the immediate or delayed test, so that each final memory test (immediate and delayed) probed the 36 original pairs, 36 perceptual lures, and 36 episodic lures, of which 24 were practiced items and 12 baseline items.

### Statistical Analyses

Statistical analyses were conducted in the same way as Experiment 1B, where in order to isolate the effects of testing on recollection, we only counted old-remember responses as correctly retrieved (Gardiner, 1988; Tulving, 1985). We first conducted a single independent *t* test on the practice benefits for original pairs (old-remember responses to practiced original pairs minus old-remember responses to baseline original pairs). This planned comparison was followed up by a 2 × 2 mixed ANOVA with factors practice type (retrieval vs. restudy; manipulated between subjects) and delay (immediate vs. delayed test; manipulated within subjects). Significant results from the ANOVA were followed up with two-tailed post hoc tests.

Although it was not our focus, we also looked at collapsed all old responses (remember and know). Results are detailed in pages 1 and 2 of the online supplemental materials. Briefly, there was a significant effect of delay and no other significant effects. These results should be interpreted with caution, given the low number of “know” responses.

Finally, we analyzed false alarms to perceptual and episodic lures by subtracting all old responses (remember and know) to lures of baseline items from old responses to lures of practiced items.

### Results

Practice effects in Experiment 2B are depicted in Figure 3B, and accuracies of practiced and baseline items are shown separately in Table 2. Our main comparison of interest yielded a significant benefit of retrieval practice for the nameable objects on the delayed test, *t*(46) = 2.02, *p* = .025. Furthermore, the 2 × 2 ANOVA revealed a significant interaction between practice and delay, *F*(1, 46) = 6.35, *p* = .015, η_*p*_^2^ = .12, and a significant main effect of delay, *F*(1, 46) = 11.66, *p* = .001, η_*p*_^2^ = .20, but no main effect of practice type, *F*(1, 46) = 0.48, *p* = .828. Post hoc *t* tests further revealed that participants in the restudy condition performed marginally better (*M* = 0.31, *SD* = 0.22) than those in the retrieval condition (*M* = 0.20, *SD* = 0.21) on the immediate test, *t*(46) = −1.79, *p* = .08, and that this pattern was reversed on the delayed test (*M*_restudy_ = 0.35, *SD*_restudy_ = 0.16; *M*_retrieval_ = 0.44, *SD*_retrieval_ = 0.16; see planned comparison). Post hoc tests, following up on the main effect of delay, showed that practice benefits were particularly evident on the delayed test, compared to the immediate test, *M*_immediate_ = 0.26, *SD*_immediate_ = 0.22; *M*_delayed_ = 0.40, *SD*_delayed_ = 0.17; *t*(47) = −3.24, *p* = .002.

Finally, when analyzing false alarm rates (old responses to lures of practiced minus of baseline items), our planned *t* test revealed no significant difference in perceptual false alarms (old responses to perceptual lures of practiced minus of baseline items) between the retrieval and restudy group on the delayed test, *t*(46) = .026, *p* = .49. There was also no significant Practice × Delay interaction, *F*(1, 46) = .181, *p* = .67, or main effect of practice, *F*(1, 46) = .132, *p* = .72. Like in Experiment 1B, there was a significant main effect of delay, *F*(1, 46) = 14.01, *p* < .001, η_*p*_^2^ = .23, evidencing that practice increased the proportion of perceptual false alarms on the delayed test (*M* = 0.09, *SD* = 0.17) relative to the immediate test, *M* = −0.04, *SD* = 0.19; *t*(46) = −3.78, *p* < .001, however, this effect was not specific to retrieval practice. Analyses on episodic false alarms (old responses to episodic lures of practiced—of baseline items), revealed no significant results, *t* test on delayed test: *t*(46) = −.65, *p* = .26; Delay × Practice interaction: *F*(1, 46) = 1.66, *p* = .20; main effect of practice: *F*(1, 46) = .097, *p* = .76; main effect of delay: *F*(1, 45) = .031, *p* = .86.

### Discussion

Using concrete nameable objects and a test where we isolate recollection-based responses, we now find the expected significant retrieval advantage at long delays, as typically reported in the testing effect literature (Coppens et al., 2011; Roediger & Karpicke, 2006b; Toppino & Cohen, 2009; van den Broek et al., 2014). Consistent with previous experiments, we also find that while restudy has an advantage at short delays, having engaged in retrieval during the practice phase entails a larger benefit when memory is tested after long delays. Thus, retrieval seems to enhance retention of visual stimuli when they can be attributed to a meaning.

For this and Experiment 1B, we had additionally hypothesized that retrieval would lead to an increase in perceptual false alarms. Across both experiments, practice led to an increase over time of perceptual, but not episodic, false alarms as evidenced by the main effect of delay when analyzing old responses to lures of practiced items minus old responses to lures of baseline items. At the delayed test, participants did commit more false alarms when judging perceptual lures (old responses to lures of practiced minus baseline items) compared to episodic lures, in line with Lee et al. (2017). However, contrary to our hypothesis, this difference was not retrieval-specific, as perceptual false alarms in the delayed test did not differ between the retrieval and the restudy groups. In our studies, therefore, retrieval-mediated learning did not specifically impair the retention of perceptual detail of the practiced images.

There are a number of possible explanations for this lack of difference in false alarm rates between practice conditions. One possibility is that the use of feedback in our studies made the retrieval condition more similar to the restudy one, reexposing participants to the visual features of the stimuli and thus leading to their enhancement. A previous study that motivated our hypothesis regarding false alarms (Lee et al., 2017) did not provide feedback. However, a direct comparison with our results is difficult because this study did not include a baseline restudy or nonpracticed condition. Another possibility is that retrieval, while boosting some aspects of the retrieved target information (including semantic information), does not actively accelerate the loss of perceptual information. Supporting this view, a recent study measuring the speed of access to different item features suggested that retrieval has a protective effect on conceptual information, with no relative difference in access to perceptual features between the retrieval and restudy groups (Lifanov et al., 2021). Together with this previous work, the present findings suggest that beyond a protective effect on semantic information, retrieval induces no active loss of perceptual detail.

## General Discussion

We here investigated under what conditions the testing effect extends to visual materials. Across four experiments, we varied the type of material (meaningless vs. meaningful visual images), and the format of the final memory test (3-AFC and remember/know recognition tests). Within each experiment, one group of participants practiced by retrieval and the other by restudy, and we measured the resulting practice benefits immediately after practice and following a 1-week delay. Our results show that retrieval practice does not enhance memory for abstract, nonmeaningful shapes irrespective of the final test format. For associations that involved target images depicting meaningful everyday objects, restudy was the more beneficial practice strategy when followed by a test immediately, however, retrieval outperformed restudy after a longer delay, in particular in Experiment 2B where we specifically probed recollection. The latter finding of a delayed retrieval advantage mirrors many studies reported in the literature using verbal materials (Roediger & Karpicke, 2006a; Rowland, 2014).

Although previous work has shown a testing effect for visual materials, it is important to emphasize that most of these studies tested participants’ memory for words or names associated with a visual stimulus, rather than its visual features (e.g., Carpenter & DeLosh, 2005; Coppens et al., 2011; Jonker et al., 2018). Furthermore, those studies that did test the retention of the visual elements of the images often used materials that can easily be verbalized (Carpenter & Kelly, 2012; Carpenter & Pashler, 2007; Guran et al., 2019; Herweg et al., 2018). As such, it is hard to distinguish if retrieval practice in these experiments enhanced the perceptual or semantic features of the stimuli. One exception is a study by Lifanov et al. (2021), which explicitly tested differential forgetting of semantic and perceptual image features. Their findings suggest that retrieval protects against the loss of semantic but not of perceptual features, in line with a semanticization account. This semantic advantage was especially evident after a 2-day delay, and significantly smaller in a restudy group. It could be argued that retrieval disproportionally strengthens semantic features because visual features decay at a faster rate (Lifanov et al., 2021; Moscovitch et al., 2016; Sekeres et al., 2016) and are not as readily available during (hence benefiting less from) retrieval practice. Consequently, the longer the delay between the initial study period and retrieval practice, the more pronounced the benefits on semantic features should become. While this explanation remains to be tested explicitly, in Lifanov et al.’s (2021) experiment, the delay between study and practice was relatively short, such that differential decay is unlikely to fully explain the pattern of results.

A study by Kang (2010) constitutes another notable exception, reporting a testing effect for difficult-to-verbalize visual stimuli. In this study, participants learned English words paired with their corresponding Chinese characters. The characters were then restudied or retrieved (visually imagined) and, in a final memory test, participants were asked to draw the Chinese character associated with each English word. Kang found a significant testing effect across three experiments, in contrast with our *squiggles* experiments (Experiments 1A and 1B). However, when Kang asked participants to describe the strategies used to perform the task (Experiment 3), 64% of strategy descriptions were classified as verbal, and the use of a verbal strategy was associated with better recall in both practice conditions. Thus, retrieval benefits in Kang’s experiments might have relied on the verbalization of stimuli, rather than the enhancement of their visual features. Although we cannot rule out that our participants relied on verbal strategies to memorize the *squiggle* images, we would not expect such a strategy to play a major role in the pattern of findings we report here. The materials were very difficult to verbalize (possibly more so than Chinese characters), and our participants did not show a testing benefit on average. Moreover, Kang’s (2010) study, as well as others showing a testing effect for visual materials (e.g., Carpenter & Pashler, 2007), assessed retrieval benefits with recall tests that have a strong generation component. In contrast, the two final test formats used in the present experiments were chosen to emphasize detailed-visual recognition to discourage the use of verbalization strategies.

Two other studies tested memory for visual image aspects, specifically, the color of the practiced stimuli (Schuetze et al., 2019; Sutterer & Awh, 2016). In both studies, participants learned nameable objects with different colors and were asked to reconstruct the original color on a color wheel, after an intervening retrieval or restudy condition. Across three experiments, Sutterer and Awh (2016) showed that retrieval practice, compared to restudy, increased the future probability of successful item access (i.e., a testing effect), but did not enhance the color precision of the recalled memories. This finding is consistent with the study by Lifanov et al. (2021) mentioned above, showing that repeated retrieval of visual object memories improves recall along with access to an image’s conceptual features but not its perceptual ones. Note that Schuetze et al. (2019) do report an additional beneficial effect of retrieval practice on participants’ confidence in their color ratings. How such measures of subjective precision relate to measures of precision derived from mixture modeling (Zhang & Luck, 2008) remains to be tested. Our own results (see the online supplemental materials) suggest that subjective confidence does not necessarily go hand in hand with objective performance measures. The studies by Lifanov et al. (2021) and Sutterer and Awh (2016) may together suggest that repeated retrieval after learning improves future access to the concept represented by a visual image, assuming that a concept includes broad categorical information (e.g., preexisting categorical color information like “red,” “blue”), but not necessarily its more subtle surface perceptual features. The findings are also in line with recent observations that reactivated memories have broader, less precise tuning curves in visual cortex than those elicited by perceived stimuli (Favila et al., 2022). Characteristics of the visual system’s architecture will therefore naturally limit the precision at which memories can be recalled, and in turn limit the precision at which retrieval can enhance previously encoded information.

The idea that retrieval-induced strengthening capitalizes on preexisting semantics is congruent with several cognitive theories of the testing effect, which all make the common assumption that retrieval’s benefits stem from a more conceptual-type processing during retrieval than during restudy. Based on the fuzzy trace theory (Brainerd et al., 2008; Brainerd & Reyna, 2004; Reyna & Brainerd, 1995), Verkoeijen et al. (2012) suggested that visual reexposure during restudy causes strengthening of surface features. In contrast, participants use semantic cues to recover mnemonic information during retrieval practice, consequently strengthening the semantic features of the memory trace. Our results are in line with this theory as we found no benefit of retrieval practice for meaningless shapes, and a type of practice that enhances surface (perceptual) features might be more beneficial for the long-term retention of these stimuli. Along similar lines, other authors (A. R. Congleton & Rajaram, 2011; A. Congleton & Rajaram, 2012; Zaromb & Roediger, 2010) suggested that restudy enhances item-specific processing, while testing enhances relational processing. A. Congleton and Rajaram (2012) showed that, compared to restudy, retrieval enhances the semantic organization of materials. With respect to our own results, retrieval advantages could thus arise from enhanced semantic organization during retrieval, and no such advantages will appear for meaningless materials that cannot be organized in taxonomic categories.

These theories of the testing effect, while well in line with our findings, do not offer a satisfying explanation of the mechanisms by which retrieval strengthens semantic over perceptual stimulus features. A possible explanation is offered by elaborative retrieval theories, which suggest that retrieval’s benefits depend on the reactivation of semantically related information during practice. This coactive information can then be used as an additional retrieval cue (Carpenter, 2009) or as mediating information that links the cue and target (Carpenter, 2011; Pyc & Rawson, 2010). The key difference between retrieval and restudy, in this framework, is that retrieval allows for related information to be coactive (and become integrated) with the target, whereas activation during restudy is largely limited to the target item itself (Antony et al., 2017; Carpenter & Yeung, 2017; Sinclair & Barense, 2019).

Evidence for the role of spreading activation during retrieval (vs. restudy) comes from both neuroimaging (Ferreira et al., 2019; Jonker et al., 2018) and behavioral (Akan et al., 2018; Chan, 2010; Mcdermott, 2006) studies. False memory studies, for instance, have shown that tests can increase the occurrence of false memories for semantically related lures, when the studied lists share a common semantic theme (Mcdermott, 2006). Moreover, several experiments have now shown that testing strengthens memory not only for the retrieved target items, but also for semantically related material encoded during initial learning, an effect termed retrieval-induced facilitation—or RIFA (Chan, 2010). RIFA is assumed to occur because people actively search for related information when attempting to retrieve the target. Critically, this effect depends on the degree to which items can be integrated—if the stimuli do not allow for integration, testing harms the retention of semantically related information, and retrieval-induced forgetting occurs instead. It is important to note that although we here focus on the reactivation of semantically related items, similar principles should apply to episodically related information, as there is evidence that retrieval can also strengthen coactivated contextual associations (Akan et al., 2018; Jonker et al., 2018; Pickering et al., 2021). Accumulating evidence thus suggests that the impreciseness of retrieval can have beneficial effects on retention (see Antony et al., 2017 for a similar argument).

Although our experiments were strongly motivated by a semanticization hypothesis, it is interesting to consider our results in light of other theories of the testing effect that do not rely on semantic spreading activation. The episodic context account (e.g., Karpicke et al., 2014) predicts a testing effect specifically on test formats that require access to contextual-episodic information. When lures and targets are all familiar or perceptually similar, as is the case in our experiments, subjects must rely on contextual information to correctly identify the target (Chang et al., 2019), potentially explaining why we found the most robust testing effects in Experiment 2B that specifically probed recollection. However, it is more difficult for the episodic context account to explain why we found a retrieval benefit for concrete object images but not for abstract shapes. It could be speculated that novel, unknown shapes become less strongly bound to the encoding context, or that their encoding context is more difficult to retrieve. However, we are not aware of empirical evidence pointing in this direction, and in fact there are theoretical models predicting stronger contextual encoding for novel materials (van Kesteren et al., 2012). As such, we believe the episodic context account cannot fully explain the present pattern of results.

A large body of evidence supports the central role of relational knowledge in retrieval-induced memory strengthening. The role that previous knowledge plays in enhancing memory retention has long been established: new information is better learned and retained if it can be integrated or contrasted with a preexisting schema (Bonasia et al., 2018; Greve et al., 2019; Schlichting et al., 2015; van Kesteren et al., 2012, 2013; Zeithamova et al., 2012). These cognitive theories are complemented by neurobiologically and computationally inspired accounts of retrieval practice effects (Detre et al., 2013; Newman & Norman, 2010; Ritvo et al., 2019) that propose that due to the imprecise nature of retrieval, the coactivation of memories is more likely to occur during testing than during restudy, where only the target pattern should be reimposed (Antony et al., 2017; Carpenter, 2009; Sinclair & Barense, 2019). Our present data suggest that previous conceptual knowledge is a necessary condition for the testing effect to emerge. Note, however, that our experiments were not initially designed for and are not sufficiently powered for cross-experiment comparisons, and we can thus only conclude that a retrieval benefit emerged for meaningful but not meaningless materials. To strengthen this conclusion, future studies should include an explicit manipulation of the type of material within the same experiment, to more directly investigate the role of previous knowledge on the presence and size of the testing effect.

In addition to the lack of a testing effect for meaningless materials, a second important finding emerged from our study: even for meaningful objects, we only found a significant testing effect when probing recollection. These results are in line with previous studies showing that retrieval boosts recollection, while leaving familiarity largely unaffected (Chan & McDermott, 2007; Pu & Tse, 2014; Verkoeijen et al., 2011). The study by Verkoeijen et al. (2011) is particularly interesting in this respect, showing that recognition decisions for previously restudied items were more familiarity-based than for previously tested ones, in addition to the recollection boost from retrieving over restudying. Recollection has been shown to reflect a more conceptual-elaborative process, while familiarity reflects a more sensory or perceptual process. For example, recollection benefits more from semantic (vs. perceptual) encoding than familiarity, whereas perceptual manipulations enhance familiarity more than recollection (see Yonelinas, 2002 for a discussion on this topic). Research on the levels of processing (Craik & Lockhart, 1972) has robustly shown that deep, semantic encoding specifically enhances recollection-based memory (e.g., Bisby et al., 2010; Bodner & Lindsay, 2003; d’Ydewalle & Van Damme, 2007; Gardiner, 1988; Gardiner et al., 1996; Java et al., 1997). According to Anderson (2020), these deep processing advantages stem from the integration of new experiences with previously stored memories. This assumption resonates well with our own findings showing the most robust testing effect on recollection-based recognition and highlights the importance of previous knowledge and semantic processing for this retrieval-induced strengthening.

A third noteworthy observation in our study was that for meaningful materials, we only found a robust testing effect after a long delay between practice and the final test. Delayed retrieval benefits are common in the testing effect literature (Coppens et al., 2011; Roediger & Karpicke, 2006b; Toppino & Cohen, 2009; van den Broek et al., 2014) and show that the behavioral benefits of retrieval evolve slowly, and are often found only after longer delays. Roediger and Karpicke (2006b), for instance, found that in an immediate test, participants performed better after studying prose passages twice than after studying and retrieving them. This pattern was reversed after 2 days, and after a week, the study plus test condition strongly outperformed studying the passages twice. Some authors suggested that the lack of an immediate testing benefit, or even a restudy benefit, is particularly pronounced when no feedback is provided during retrieval practice (Rowland & DeLosh, 2015), consistent with a bifurcation explanation (e.g., Kornell et al., 2011; van den Broek et al., 2014; but see Carpenter, 2011; A. Congleton & Rajaram, 2012 for alternative explanations of the Practice × Delay interaction). According to this view, retrieval practice significantly strengthens only the subset of items that can be successfully retrieved during practice, while restudy moderately and more uniformly strengthens all practiced items. Forgetting over time then pushes most restudy items below the accessibility threshold, while the subset of items that were successfully retrieved during practice will remain accessible even after longer delays. Feedback should, in theory, attenuate a bifurcation of the item distribution. In Experiment 2B, however, we observed a Practice × Delay interaction despite giving feedback. It is possible that the large number of arbitrary word–image associations used in our experiments resulted in relatively low item strength even with feedback provided. Overall, too few studies currently exist that use visual materials and test memory over various delays, making it difficult to draw conclusions about the robustness of this Practice × Delay interaction for visual stimuli.

In summary, we show across four behavioral experiments that testing effects for visual stimuli rely on existing knowledge and are most robustly found at long delays and when probing the recollective component of memory. Novel, meaningless shapes instead might benefit from repeated visual exposure, especially when using a visually driven final memory test. Together, our findings inform cognitive and neurobiologically inspired theories of the testing effect, supporting those that place an emphasis on the role of spreading activation during retrieval. They also have important implications for educational contexts, showing that the most effective rehearsal strategies depend on the type of to-be-remembered material.

## Supplementary Material

10.1037/xlm0001248.supp

## Figures and Tables

**Table 1 tbl1:** Proportion of Correct Responses (Experiment 1A) and of Correct “Old-Remember” Responses (Experiment 1B), as Well as Practice Effects for Each Condition Across the Two Experiments

Item type	Experiment 1A		Experiment 1B	
	Immediate, *M* (*SD*)	Delayed, *M* (*SD*)	Immediate, *M* (*SD*)	Delayed, *M* (*SD*)
Retrieval				
Practiced	0.83 (0.17)	0.63 (0.21)	0.64 (0.22)	0.37 (0.28)
Baseline	0.76 (0.20)	0.65 (0.18)	0.44 (0.26)	0.21 (0.29)
Practice effects	0.07 (0.05)	−0.02 (0.18)	0.20 (0.21)	0.16 (0.24)
Restudy				
Practiced	0.86 (0.12)	0.66 (0.19)	0.74 (0.20)	0.32 (0.25)
Baseline	0.74 (0.24)	0.54 (0.24)	0.45 (0.27)	0.14 (0.18)
Practice effects	0.12 (0.23)	0.12 (0.24)	0.29 (0.22)	0.18 (0.15)
*Note*. Mean (standard deviation).				

**Table 2 tbl2:** Proportion of Correct Responses (Experiment 2A) and of Correct “Old-Remember” Responses (Experiment 2B), as Well as Practice Effects for Each Condition Across the Two Experiments

Item type	Experiment 2A		Experiment 2B	
	Immediate, *M* (*SD*)	Delayed, *M* (*SD*)	Immediate, *M* (*SD*)	Delayed, *M* (*SD*)
Retrieval				
Practiced	0.87 (0.11)	0.54 (0.19)	0.81 (0.20)	0.57 (0.20)
Baseline	0.76 (0.20)	0.36 (0.18)	0.61 (0.23)	0.13 (0.11)
Practice effects	0.11 (0.16)	0.18 (0.14)	0.20 (0.21)	0.44 (0.16)
Restudy				
Practiced	0.92 (0.08)	0.57 (0.16)	0.89 (0.11)	0.51 (0.20)
Baseline	0.75 (0.16)	0.43 (0.18)	0.58 (0.24)	0.16 (0.14)
Practice effects	0.17 (0.13)	0.14 (0.14)	0.31 (0.22)	0.35 (0.16)
*Note*. Mean (standard deviation).
