# Supplementary material for: The Testing Effect for Visual Materials Depends on Preexisting Knowledge
Source: J Exp Psychol Learn Mem Cogn. 2023 Jun 8;49(10):1557–71. doi: 10.1037/xlm0001248 (PMC10519161; doi:10.1037/xlm0001248)
Supplement: Supplementary file 1 [file xlm0001248_sm.docx]

**SUPPLEMENTARY MATERIALS**

**Old-know responses**

We conducted statistical analyses both for “old-know” responses separately and collapsing all remember responses (remember and know), using the same logic as in the main manuscript: performing a targeted 1-tailed t-test first, comparing practice effects (practiced-baseline items) in the retrieval and restudy groups and then conducting a 2x2 ANOVA to assess a potential practice by delay interaction. We first report the results for Experiment 1B (squiggles) and then for experiment 2B (nameable objects).

It should, however, be noted that the amount of old-know responses was too low to allow for the separate analyses to be robust. Across the two experiments, participants generally gave an old-know response for original pairs in less than a quarter of trials (See Supplementary Table 1).

*Experiment 1B*

For old-know responses, there was no significant benefit of practice on the delayed test (t(45) = 0.18, p =.49). The 2x2 ANOVA yielded a significant main effect of delay (F(1,45) = 9.92, p=.003), but no main effect of practice (F(1,45) = .042, p=.84), nor an interaction between the two (F(1,45) = .06, p = .81). Post-hoc tests, following up on the main effect of delay, showed that practice benefits were particularly evident on the delayed test, compared to the immediate test (M_immediate_ = -.11, SD_immediate_ = 0.20; M_delayed_ = 0.14, SD_delayed_ = 0.20; t(46) = -3.18, p = .003).

Collapsing old-remember and old-know responses, there was still no significant effect of practice (t(45) = -.18, p = .43). Furthermore, the ANOVA yielded no significant results (main effect of practice: F(1,45) = 1.49, p=.23); main effect of delay F(1,45) = 1.18, p=.28; delay x practice interaction F(1,45) = .99, p=.32).

*Experiment 2B*

Analysing old-know responses only, the targeted *t*-test revealed no significant differences between retrieval and restudy on the delayed memory test (t(46) = -.16, p = .44). ANOVA results showed no significant practice x delay interaction (F(1,46) = 2.95, p = .09), nor significant main effects (delay: F(1,46) = 2.68, p = .11; practice: F(1,46) = 1.51, p=.23).

When collapsing old-remember and old-know responses, no significant differences arose on the final test between the two practice conditions (t(46) = .13, p = .10). Similarly, no significant main effect of practice (F(1,46) = .90, p = .35) or delay x practice interaction (F(1,46) = 1.14, p = .291) were found in the 2x2 ANOVA. However, there was a significant main effect of delay (F(1,46) = 25.54, p=.000). Practice effects were higher in the delayed memory test (M = .27, SD = .22) compared to the immediate test (M = .17, SD = .19; t(46) = -5.05, p < .001).

**Supplementary Table 1:** Proportion of “old-know” responses in Experiments 1B and 2B for practiced and baseline items (across conditions) on the immediate and delayed tests. Mean (standard deviation).

|  |  | Test | |
| --- | --- | --- | --- |
|  |  |  |  |
|  |  | Immediate  M(SD) | Delayed  M(SD) |
| *Experiment 1B (squiggles)* |  |  |  |
| Practiced |  | 0.17 (0.17) | 0.22 (0.16) |
| Baseline |  | 0.28 (0.23) | 0.21 (0.18) |
|  |  |  |  |
|  |  |  |  |
| *Experiment 2B (Objects)* |  |  |  |
| Practiced |  | 0.09 (0.15) | 0.21 (0.16) |
| Baseline |  | 0.17 (0.19) | 0.24 (0.23) |

**Subjective judgments**

*Retrieval practice*

To further equate the retrieval and restudy conditions, in Experiments 1B and 2B, we asked participants in the retrieval condition not only to think back to the correct image, but also to press a key to indicate whether they thought they remembered the correct stimulus or not (note that participants in the restudy condition already had to press an arrow key to indicate whether they found it easy or hard to link the word and the image). This provided us with a subjective measure of retrieval practice success.

In both experiments, participants in the retrieval condition reported remembering more stimuli in the second cycle of practice compared to the first and remembering at least 50% of the items in the second repetition (Supplementary Table 2).

|  |  | Remember  M(SD) | Don’t remember  M(SD) |
| --- | --- | --- | --- |
| *Experiment 1B (squiggles)* |  |  |  |
| Repetition 1 |  | 0.47 (0.17) | 0.50 (0.17) |
| Repetition 2 |  | 0.54 (0.25) | 0.44 (0.25) |
| Overall |  | 0.50 (0.20) | 0.47 (0.20) |
|  |  |  |  |
|  |  |  |  |
| *Experiment 2B (Objects)* |  |  |  |
| Repetition 1 |  | 0.55 (0.13) | 0.44 (0.13) |
| Repetition 2 |  | 0.79 (0.12) | 0.21 (0.11) |
| Overall |  | 0.67 (0.11) | 0.32 (0.11) |

**Supplementary Table 2:** ‘Remember’ and ‘Don’t remember’ responses during the practice phase (repetitions 1 and 2) for participants in the retrieval condition in Experiments 1B (squiggles) and 2B (objects).

*Final test*

Before responding to the stimuli in the final tests (that is, deciding which image went with a specific word cue in the 3-AFC experiments or deciding whether a pair was old or new), participants were asked to make a subjective judgment upon seeing the cue word. Participants were instructed to press a key to indicate whether they thought they remembered the correct item or not. We next report the results of these subjective judgments in the form of practice benefits (remember responses to practiced items – remember responses to baseline items) to explore, purely descriptively, if they mirror the results from objective judgements reported in the main manuscript.

Experiment 1A

Subjective judgments in this experiment show a different pattern than the objective measure in the final test. Subjective judgments show what would be the typical pattern found in testing effect studies: a restudy advantage on the immediate test (M_retrieval_ = 0.17, SD_retrieval_ = 0.19; M_restudy_ = 0.30, SD_restudy_ = 0.21), but a retrieval advantage on the delayed test (M_retrieval_ = 0.33, SD_retrieval_ = 0.16; M_restudy_ = 0.18, SD_restudy_ = 0.23). Thus, while participants that had performed retrieval practice felt more confident in their memory for practiced items on the delayed test, they objectively showed reduced retrieval benefits compared to participants in the restudy condition.

Experiment 1B

For this experiment, we analysed subjective judgments to each cue word, regardless of item type (original pair, perceptual or episodic lure), since the subjective judgment is made before the target and lure items appear on the screen and is thus unrelated to lure type.

In this experiment, subjective judgments from participants in the restudy condition showed a numerical practice advantage compared to retrieval, in both the immediate (M_retrieval_ = 0.20, SD_retrieval_ = 0.17; M_restudy_ = 0.26, SD_restudy_ = 0.18) and the delayed tests (M_retrieval_ = 0.14, SD_retrieval_ = 0.23; M_restudy_ = 0.18, SD_restudy_ = 0.20).

Experiment 2A

A similar pattern to Experiment 1A was found in Experiment 2A. Whereas in the immediate test, participants in the restudy condition judged their memory for practiced items better than participants in the retrieval condition (M_retrieval_ = 0.30, SD_retrieval_ = 0.31; M_restudy_ = 0.36, SD_restudy_ = 0.26), this pattern was reversed (although with a very small difference between conditions) on the delayed test (M_retrieval_ = 0.07, SD_retrieval_ = 0.22; M_restudy_ = 0.05, SD_restudy_ = 0.16). This pattern mirrors the one observed in the final tests’ objective measures.

Experiment 2B

Subjective judgments in this experiment show that while participants in the retrieval and restudy condition were equally confident in their success in the immediate test (M_retrieval_ = 0.27, SD_retrieval_ = 0.14; M_restudy_ = 0.28, SD_restudy_ = 0.19), participants’ judgments in the retrieval condition show a greater practice advantage than in the restudy condition on the delayed test (M_retrieval_ = 0.32, SD_retrieval_ = 0.16; M_restudy_ = 0.18, SD_restudy_ = 0.17), in line with objective practice benefits.
